# The Role of Skin-to-Skin Contact and Breastfeeding on Atonic Postpartum Hemorrhage

**DOI:** 10.3390/nursrep11010001

**Published:** 2020-12-25

**Authors:** Wedad M. Almutairi, Susan M. Ludington, Mary T. Quinn Griffin, Christopher J. Burant, Ahlam E. Al-Zahrani, Fatmah H. Alshareef, Hanan A. Badr

**Affiliations:** 1 Maternity and Child Department, Faculty of Nursing, King Abdulaziz University, Jeddah 21551, Saudi Arabia; aealzahrani@kau.edu.sa (A.E.A.-Z.); habadr@kau.edu.sa (H.A.B.); 2 Bolton School of Nursing, Case Western, Reserve University, 10900 Euclid Avenue, Cleveland, OH 44106-4904, USA; sml15@case.edu (S.M.L.); mtq2@case.edu (M.T.Q.G.); cxb43@case.edu (C.J.B.); 3 Medical Surgical Department, Faculty of Nursing, King Abdulaziz University, Jeddah 21551, Saudi Arabia; falsharif@kau.edu.sa

**Keywords:** skin-to-skin contact (SSC), breastfeeding (BF), postpartum hemorrhage (PPH)

## Abstract

*Objectives:* were to (a) determine incidence of postpartum hemorrhage (PPH) in all women delivering between 2009 and 2015, and (b) determine the amount of Estimated Blood Loss (EBL) and duration of the third stage of labor in each subgroup for women with or without PPH, and (c) compare EBL and duration of 3rd stage of labor between subgroups in groups of women with or without PPH. *Design:* A retrospective chart review conducted using codes for atonic PPH. *Setting:* Records from a University based tertiary setting, 264 charts were reviewed and data from 154 charts were analyzed. One-way ANOVAs followed with post-hocs and a 2-way ANOVA were conducted. *Results:* PPH rate increased by 47.50% from 2009–2015. For women with PPH, EBL was lower in skin to skin contact (SSC) + Breastfeeding (BF) subgroup. For women without PPH, EBL was lower in SSC only subgroup. Third stage of labor duration was longer in women with PPH. *Conclusions:* Study confirmed the increasing trends of PPH due to uterine atony and proposed role of SSC and BF in decreasing EBL and shorten the duration of the 3rd stage of labor for PPH women, usefulness of SSC and BF as physiologic practices merit further study.

## 1. Background

An estimated of 295,000 maternal deaths occurred worldwide in 2017 (PPH) [1]. Postpartum Hemorrhage (PPH) continues to be the number one cause of maternal death around the globe, and it is mostly caused by uterine atony [2,3] even though PPH is a preventable and treatable condition [2].

The PPH is defined as estimated blood loss (EBL) greater than 500 mL in a vaginal delivery and more than 1000 mL in a cesarean delivery [1]. The third stage of labor is defined as the period from delivery of the newborn to expulsion of the placenta and membranes. The prolonged third stage of labor is the strongest predicting factor for PPH [4,5]. The prolonged third stage of labor has been defined as a duration longer than 30 min since 1991 [6]. However, a 2016 cohort study of 7121 women suggested that the risk of PPH increases when the duration of the third stage of labor is 20 min or longer [4]. An updating prospective study including 600 women stated that if duration of third stage of labor >15 min, women 15.5 times have higher risk of PPH [7].

As a preventable condition, PPH has posed a challenge to obstetric care worldwide [8]. Over the last two decades, PPH has increased significantly in all developed countries [2,9]. In the United States alone, PPH increased by 27.5% from 1995–2004 and represented 19.1% of all hospital deaths [10]. The increase is primarily due to uterine atony [8]. Severe PPH due to uterine atony has doubled from 1999–2008 [11]. As of 2012, the rate of PPH in the United States had not changed from the 2010 data; overall the incidence of severe PPH doubled from 2001/2002 to 2011/2012 [12].

Researchers have proposed that one contributor to the increasing rate of PPH due to uterine atony in developed countries may be medicalization of birthing process, which is the increased use of unnecessarily medical interventions during the normal birth [13] rather than fostering physiologic processes of labor and delivery that mammals have practiced for millennia, especially keeping the newborn in close contact with the mother as often as possible and allowing breastfeeding within minutes of birth [14].

Empirical studies suggested that the prophylactic administration of exogenous oxytocin, may increase the risk of PPH due to uterine atony [2] because of the desensitization of uterine receptors due to overstimulation with oxytocin that lead to uterine muscle exhaustion which in turn cause uterine atony, desensitization of oxytocin receptors has been evident in a recent in vitro study that examines the contractile response of human pregnant myometrium to oxytocin after pretreatment with oxytocin and the results revealed that pretreatment with oxytocin decrease the oxytocin-induced uterine contractions due to receptors desensitization phenomena [15].

The current prevention interventions for atonic PPH are predominantly medical and pharmacological methods that constitute Active Management of Third Stage of Labor (AMTSL). AMTSL consists of administering exogenous oxytocin, early cord clamping, and controlled cord traction to facilitate placental delivery and prevent PPH [16]) AMTSL aims to enhance uterine contractility and shorten the third stage of labor. Non-pharmacologic treatments also exist to prevent PPH. Uterine massage has been a common intervention, but sustained uterine massage is a poorly validated intervention that is no longer recommended [17].

Midwives use AMTSL but often substitute expectant management of the third stage of labor to make labor “normal” without excessive medical interventions [18,19]. Expectant management means a “hands-off” policy, which includes waiting for the signs of placental separation and allowing the placenta to deliver spontaneously. Midwives have added psychophysiological care to expectant management so mothers feel connected to their environment, safe, secure, and attended during labor [14].

Components of psychophysiological care include immediate and sustained skin-to-skin (e.g., chest-to-chest) contact between the mother and newborn and immediate breastfeeding during the third and fourth stages of labor [20] Hastie and Fahy (2009) have reported that midwives consider the absence of skin-to-skin contact to be a risk factor for PPH [21]. Thus, both skin-to-skin contact and breastfeeding have been tested as physiologic components of psychophysiological theory [19] which is also known as the pronurturance theory [14]), and have been deemed to be especially important during the first two hours’ post-birth to prevent PPH [20].

Saxton et al. analyzed all 7548 records of women who delivered in a tertiary hospital in Australia under care of equal numbers of physicians and midwives who use limited amounts of exogenous oxytocin. They found that women who did not have SSC and BF were twice as likely to have PPH than women who had SSC and BF within 30 min of birth, but they did not measure EBL or the duration of the third stage of labor in women with PPH [20].

Therefore, additional testing of EBL and the duration of the third stage of labor is needed, moreover, data are resulting from Australia, which has as different birth practices compare to United States. Because there is insufficient evidence regarding the roles of SSC and BF in reducing PPH in the United States, we conducted a retrospective chart review dividing the data into two groups (i.e., women with PPH and women without PPH) and four subgroups (i.e., SSC only, BF only, SSC + BF, and no SSC/no BF). The objectives were to (a) determine the incidence of PPH in all women delivering between 2009 and 2015, (b) determine the amount of EBL and duration of the third stage of labor in each subgroup for women with or without PPH, and (c) compare EBL and duration of third stage of labor between subgroups (i.e., SSC only, BF only, SSC + BF, and no SSC/no BF within the first 2 h post-birth) in women with or without PPH from 2009–2015 at a university hospital.

## 2. Methods

### 2.1. Study Design

We conducted a retrospective comparative chart review by extracting data from electronic medical records coded as ICD9-666.0, ICD9-666.1, ICD10-072.0, and ICD10-072.1.1 for women with atonic PPH and records without complication codes for women without PPH from 2009–2015. We obtained university Institutional Review Board (IRB) approval (UHCMC IRB number: 06-16-15).

### 2.2. Setting

All maternity records from a University-based tertiary maternity hospital from 2009 through 2015 were reviewed on encrypted computers in a confidential setting in the hospital.

### 2.3. Ethical Consideration

Institutional review Board (IRB) approval obtained from the UH hospital IRB. Because the study is a retrospective study that used preexisting data, all aspects of human subject protection may not apply. There was no known harm to subjects by conducting the retrospective chart review because data were de-identified and there was no links to other identifiable data such as names, medical record numbers, birth dates, or social security numbers. Data related to subjects in the current study used for scientific purpose only. Access to data is restricted for authorized personnel. An identification number assigned for each record of data with a link to the electronic medical record number. The master sheet that include the EMR# of the subjects was kept in the P. drive in the UH hospitals computers only. Only the PI and UH IRB authorized personnel have access to the master sheet linking subject ID with electronic medical record number. Signed consent is not relevant for a de-identified retrospective chart review study.

### 2.4. Participants

Inclusion criteria for women’s medical records in the PPH group were (a) only PPHs due top uterine atony that occurred in the first 24 h post-birth, plus the following inclusion criteria for PPH and without-PPH group medical records: (b) mother delivered a term or near term newborn (36–42 weeks gestation) because term infants are available to the mother for immediate placement in SSC (American Academy of Pediatrics, 2002), (c) maternal age was between 19–60 years, (d) women with any BMI, and (e) history of cesarean section and/or previous PPH were included. Exclusion criteria were (a) women who had a documented diagnosis of chorioamnionitis, placenta abruptio, placenta previa, and any placental abnormalities during the antenatal period, (b) women who experienced a stillbirth, (c) delivery of premature (<37 weeks) or post-mature (>42 weeks) infant, and (d) newborn with low APGAR (<7 at 5 min) score.

Two lists of Electronic Medical Records Number (EMRN) in XL format for patients who had PPH codes from 2009–2015 and for patients who had an institution-based code for uncomplicated birth were received following IRB approval. Each chart was opened on a hospital computer in a specified secure and restricted space so the computer screen could not be seen by anyone else. We recorded de-identified extracted data on the Data Collection Sheet. Each subject had a separate data collection sheet. The number of all records constituted the data for number of women with PPH each year. In addition to the list of records with the code for PPH, a list of women without PPH was also generated. The records from each year were sequentially read according to the group (PPH and without-PPH) and to subgroup based on treatment (SSC, BF, SSC + BF, no SSC/no BF) documented in the chart.

### 2.5. Variables

The main variables of the study were the number of diagnoses of PPH each year, duration of the 3rd stage of labor in minutes, estimated blood loss in milliliters (mLs), and treatments within the groups (SSC only, BF only, SSC + BF, No SSC/No BF).

The operational definitions of the variables are as the following: (a) PPH is the number of cases that had the following codes: ICD9-666.0, ICD9-666.1, ICD10-072.0, and ICD10-072.1.1 in the Electronic Medical Records, (b) duration of the 3rd stage of labor is the presence of documentation in the mother’s medical record of the duration of third stage of labor in minutes and/or hours, (c) EBL is the presence of the documentation of the number of mLs of blood loss in the labor and delivery flow chart, (d) BF only is the presence of documentation in the mother’s medical record that the newborn fed at or latched onto the breast means the infant’s mouth was on the nipple within first two hours post-birth, (e) SSC only the presence of any documentation that the newborn was placed in SSC immediately or within the first two hours post-birth, (f) SSC + BF is the presence of the documentation of both SSC and BF immediately or within the first two hours post-birth, and (g) No SSC/No BF is the absence of the documentation of SSC and BF immediately or within the first two hours post-birth.

### 2.6. Data Sources and Managment

We created a Data Collection Sheet consisting of 33 items related to demographic (e.g., maternal age and ethnicity), maternal (e.g., gravidity, parity, weeks of pregnancy, history of blood disorders, history of previous PPH, type of delivery, length of third stage of labor, estimated amount of blood loss), and newborn (e.g., birth weight, APGARs) medical data, which we submitted to three experts who confirmed the content’s validity. The variables were the groups (i.e., women with PPH and women without PPH), subgroups (i.e., SSC only, BF only, SSC + BF, and no SSC/no BF). The variables of interest were number of women with PPH by year, EBL (mLs), and duration of the third stage of labor (minutes).

All extracted data (hard copies) from the study saved in a locked cabinet in a locked office in the School of Nursing building where only the PI, IRB and School of Nursing personnel have access to the materials as authorized and permitted. All electronic data were saved in a password-protected computer and coded so that only the researchers were able to access the data.

### 2.7. Bias

Bias in data collection was controlled by having only one primary extractor which minimized discrepancies in data recording decisions and optimizing the validity of the study (Matt and Matthew, 2013), and accuracy of data extraction was verified by having the second author review the data on the data collection sheet while the first author reread the data from the medical record.

### 2.8. Study Size

For the first objective, a power analysis was not conducted because the goal was to identify the frequency of women having atonic PPH over the 6 years. A Cohen (Cohen, 1992) power analysis with alpha level of 0.05, power of 0.80, and medium effect size (0.30), revealed that 120 total records per group were needed to have a fully powered ANOVA; 264 charts were reviewed but documented subgroup data were available in only 154 charts. However, after conducting the study, the SSC only and BF only subgroups constituted very few records.

### 2.9. Quantitative Variables

Quantitative ratio variables (age, EBL, duration of 3rd stage of labor) were analyzed measures of central tendency (Mean, Minimum, Maximum), and dispersion (SD); quantitative categorical variables were measured by frequencies and percentages. Differences between subgroups and groups were by ANOVA and two-way ANOVA with post-hoc tests.

### 2.10. Statistical Methods

Data were transcribed from the Data Collection Sheet into SPSS 22.0 for analysis. For the incidence of PPH over 6 years and each year, the absolute number of PPH cases per year was recorded and then summed for a total. To compare the subgroups in each group on each outcome (EBL and duration of 3rd stage of labor), a one-way ANOVA with a Bonferroni post-hoc test was conducted. Two Two-Way ANOVAs for each outcome (EBL, duration of 3rd stage of labor) to examine the interactions between the groups and subgroups were also conducted. The interaction based on a Two-Way ANOVA helps us to understand whether the effect of each subgroup, i.e., intervention (for example, SSC only) on the estimated blood loss and duration of 3rd stage of labor was the same or different in the two groups (women with PPH, women without PPH) by illustrating the pattern of intervention (subgroups) effects on estimated blood loss and duration of 3rd stage of labor for the two groups. The BF-only subgroup was omitted from inferential analyses for both groups due to its small sample size; the no SSC/no BF subgroup data for women without PPH were also omitted in the analysis of the duration of the 3rd stage of labor due to its small sample size—documented duration data were only available in three charts. Thus, charts with missing data were deleted from analyses.

## 3. Results

### 3.1. Participants

In total, 1480 charts for women with atonic PPH were retrieved and 1500 charts for women without PPH were retrieved. Then, 265 charts, 155 charts for women with atonic PPH and 110 charts for women without PPH were reviewed. Of the 265 charts reviewed, 52 were eliminated for failure to meet inclusion criteria (19.62%) and 59 (22.30%) for incomplete charting. The final sample size eligible for analysis was 154 charts, 79 (51.3%) for women with PPH diagnosis, and 75 (48.7%) for women without PPH diagnosis. The total number of charts for each subgroup for both women with and without PPH is listed in Table 1.

### 3.2. Descriptive Data

The mean age of women with PPH was 28.82 (SD = 6.63) and of women without PPH was 26.67 (SD = 4.42). Among the women with PPH, 70.9% were African American, 24.1% were White, and 1.3% were of another race; among the women without PPH, 65.3% were African American, 32% were White, and 1.3% were of another race. Maternal and infant medical data are depicted in Table 2.

### 3.3. Outcome Data

#### 3.3.1. Incidence of PPH

Incidence of PPH due to uterine atony increased from 2009 to 2015 by 47.50%. Table 3 illustrates the rate per year from 2009 until 2015.

#### 3.3.2. Duration of 3rd Stage of Labor

The lengths of the 3rd stage of labor (in minutes) for each subgroup were described and illustrated in Table 4. The duration of the 3rd stage of labor appeared to be longer in the PPH group than in the non-PPH group which is expected. The shortest duration of 3rd stage was in the SSC + BF subgroup of women with PPH diagnosis. However, one-way ANOVAs revealed no significant differences in the duration of the 3rd stage of labor between the subgroups in the group of women with PPH (*F* (*2, 65*) = 1.46, *p* = 0.24) and in the group of women without PPH (*F* (1, 37) = 0.02, *p* = 0.89).

#### 3.3.3. Estimated Blood Loss

The descriptive data about EBL for each subgroup in women with and without PPH are in Table 5. In both groups (PPH group and without PPH (normal), the SSC + BF subgroup had the lowest EBL values. For women with PPH, one-way ANOVAs revealed EBL differed among subgroups, *F* (2, 70) = 4.37, *p* = 0.02. The Bonferroni post hoc test revealed that EBL in the no SSC/no BF subgroup was higher than in the SSC + BF subgroup (*p <* 0.05). For women without PPH, EBL also differed among subgroups, *F* (2, 67) = 3.598, *p* = 0.033, with the Bonferroni post hoc test showing that EBL was higher in the no SSC/no BF subgroup than in the SSC-only subgroup (*p <* 0.05).

### 3.4. Other Analyses

#### Two-Way ANOVA Results

The grouping effect on the duration of the 3rd stage of labor was significant (*F* (1, 100) = 5.89, *p* = 0.02, Partial Eta Squared = 0.1), indicating that women with PPH had an approximately 10-min longer duration of the 3rd stage of labor than women without PPH. A significant interaction between the groups and the subgroups regarding EBL (*F* (2, 137) = 3.72, *p* = 0.03) was also found, indicating that women with PPH benefitted more than women without PPH from the treatment of SSC + BF. The estimated marginal mean showed that women with PPH in the SSC + BF subgroup had approximately 400 mL less EBL than women with PPH in the No SSC/No BF subgroup. For women without PPH estimated EBL difference between SSC + BF and No SSC/No BF subgroups was about 100 mLs.

## 4. Discussion

### 4.1. Key Results

A retrospective chart review study revealed that the answer to the first objective was that atonic PPH rate increased by 47.5% between 2009–2015. In regard to the second objective, for women with PPH, SSC only, SSC + BF, No SSC/No BF subgroups had a duration of 3rd stage of labor that was >10 min, with the longest duration (18 min) being in the No SSC/No BF subgroup.

In regard to the third objective, women with PPH who received SSC + BF had significantly less EBL than women who had No SSC/No BF. For women without PPH, those in the SSC only subgroup had significantly less EBL than women in the No SSC/No BF subgroup. An additional finding was that being in the SSC + BF subgroup significantly benefitted women with PPH more than it benefitted women without PPH (normal).

### 4.2. Interpretation

#### 4.2.1. Incidence of PPH

The increasing rate of atonic PPH found is similar to Callaghan et al.’s U.S. [22]. study that found PPH due to uterine atony increased by 50% from 1994 to 2006. and Maswime and Buchmann systematic review [23]. The increase reported here was higher than what was reported in a British study in which atonic PPH increased by 33% from 2000–2009 [24]. One explanation for the increasing incidence of PPH in the setting hospital could be the 2012 policy that uterine atony be routinely treated with 30 units of prophylactic Pitocin (an exogenous synthetic oxytocin). The emergence of increased prophylactic doses of exogenous oxytocin to prevent PPH led to the ACOG’s 2015 recommendation to use 10–40 units of Pitocin to actively manage the 3rd stage of labor. A case control study in the United States of 108 women with and without atonic PPH found that women had a 58% greater risk for PPH due to uterine atony for every 5000 milliunit dose of Pitocin they received [25]. A population-based cohort study conducted in Australia found strong association between oxytocin infusion during labor and sever PPH [26], which was confirmed in a Canadian case control study conducted from 2011 until 2013 [1] and secondary analysis cross sectional study from WHO survey data [27]. These studies support the hypothesis of medicalization of the normal birthing process in developed countries, and may be one explanation for the increasing rate of atonic PPH [13,28].

Another explanation for the increased incidence of PPH due to uterine atony is the scientific evidence of desensitization of oxytocin receptors due to the persistent stimulation by synthetic oxytocin, which in turn leads to suppressed oxytocin-induced contractility in the uterine myometrium, resulting in uterine atony [15]. Minimizing unnecessary interventions such as high dosages of Pitocin, was tested in a pretest-posttest evaluation of an educational program about unnecessary routine interventions during labor and delivery. The educational program resulted in significantly reduced oxytocin dosages without adverse events [29].

#### 4.2.2. EBL

Women with PPH who received SSC + BF had less EBL than women with PPH in the No SSC/No BF subgroups. Women without PPH in the SSC only subgroup had less EBL than women in No SSC/No BF subgroups. Our finding of less EBL in women who had SSC + BF is similar to that of a randomized controlled trial of 216 women who did SSC during the 3rd stage of labor and experienced lower blood loss as measured by sanitary napkin use than 216 mothers who did not experience SSC + BF [30]. A quasi-experimental study revealed that 100% of women who experienced normal births and did SSC during the 3rd stage of labor had no uterine atony nor excessive EBL, but 28% of women who did not do SSC had uterine atony and excessive EBL [31]. Unfortunately, Essa et al. and Dordevic et al. did not measure SSC + BF nor did they quantify the estimated or actual blood loss, making comparisons to their studies difficult.

Three explanations for decreased EBL in women who received SSC + BF exist. First, the decreased EBL was most likely due to SSC [32] and BF [33] which, independently and in combination [20] increase the level of endogenous oxytocin in the mother’s blood. Handlin et al. quasi-experiment with 63 primiparous women who did SSC found that SSC lowered cortisol and adrenocorticotropin hormone (ACTH) levels in maternal blood, lowered cortisol and ACTH, and increased the circulating endogenous oxytocin levels [33]. Increased levels of endogenous oxytocin cause effective contractions of the myometrium and occlude blood vessels, preventing blood loss [20]. A second explanation may be the difference between the synthetic oxytocin medically administered and the endogenous oxytocin produced by SSC and BF. Endogenous oxytocin released during SSC + BF has the ability to cross the blood and brain barrier and enhance the sensitivity of oxytocin receptors for better functioning, but the synthetic oxytocin does not cross this barrier. The third explanation may be that SSC and BF, especially within the first 30 min post-birth, provide a physiologically supportive and nurturing environment for the mother [14]. A nurturing environment for the mother occurs when SSC and BF reduce maternal stress by increases circulating levels of endogenous oxytocin [14]. When physiologically supportive care from SSC and BF have been provided, increased endogenous oxytocin decreased the risk of PPH by 50% [20].

#### 4.2.3. Duration of 3rd Stage of Labor

For women with PPH group. Women with PPH experienced >10 min durations of the 3rd stage of labor. This finding was expected because the longer duration of the 3rd stage of labor is a predictor of PPH due to uterine atony [4]. Recently, a duration of 20 min or more has been identified as critical to the prediction of complications [4] therefore, reducing the duration of the 3rd stage of labor is clearly a preventative strategy for PPH [7].

### 4.3. Additional Finding

The SSC + BF treatment had stronger impact on the EBL for women with PPH than women without PPH. Thus, women with PPH benefited more. Our data suggest that even with the higher dose of synthetic oxytocin administered to women with PPH, SSC + BF may be able to assist the system so that the negative impact of high doses of synthetic oxytocin can be minimized and can be used as an adjunct therapy to reduce severity of blood loss in women with PPH.

### 4.4. Limitations

One limitation of the study was inaccurate documentation in the patients’ medical records, which is difficult to correct. Inappropriate coding of PPH, which resulted in some women having PPH codes, even though their EBLs were less than 500 mL, also occurred. We found 11 cases representing incorrect coding in the 155 retrieved records. Another limitation was the presence of missing data in many of the records. Missing data routinely occurs in chart review studies and is often found routinely in any chart (The Editors of The Lancet, 2017). Another limitation was small sample size for two subgroups (SSC only and BF only). For example, the BF only subgroup in both groups and the No SSC/No BF subgroup in women without PPH group were deleted from analyses.

Though limitations exist, the study also had strengths and importance. The study is the first to describe the role of SS and BF on the duration of the 3rd stage of labor and EBL in women with and without PPH. In addition, the data can be used to inform health care providers to anticipate applications of low-cost interventions like SSC, BF, and SSC + BF, such as those being encouraged by the Maternal Hemorrhage Quality Improvement Collaborative (Lydon and Cape, 2016. The clinical implications of this study are to (a) increase nurses and physicians’ awareness of the potential effects of early SSC, BF, and SSC + BF in parturient women, especially those at high risk of PPH; (b) reinforce the practice of SSC, BF, and SCC + BF as an element of Essential Care of Every Newborn [18,19]; and (c) understand the need to enhance providers’ knowledge and skills in relation to SSC and BF practices through continuing education.

### 4.5. Generalizability

Because our sample was mostly African American and the study was conducted in one setting only, caution must be exercised when considering the generalizability of our data. Additionally, caution must be practiced because of the retrospective chart review study design.

In conclusion, the findings revealed that the combination of SSC + BF may have a positive impact on the duration of third stage of labor and estimated blood loss for women with PPH and women without PPH. The results have implications for maternity practices worldwide. PPH remains the primary source of maternal death and a critical target for reducing maternal mortality [1].

## Figures and Tables

**Table 1:** Number of Charts Reviewed for Each Subgroup of the Total Sample.

Subgroups	Total Number and Percentage of Charts *n* = 154	Number and Percentage of Charts for PPH Women *n* = 79	Number and Percentage of Charts for Non-PPH Women *n* = 75
SSC only	18 (11.7)	11 (13.9%)	7 (9.3%)
BF only	7 (4.5)	4 (5.1%)	3 (4.0%)
SSC + BF	60 (39.0)	29 (36.7%)	31 (41.3%)
No SSC/No BF	69 (44.8)	35 (44.3%)	34 (45.3%)

**Table 2:** Maternal and Infant Medical Data for Women with/without PPH.

Groups	Characteristics	Mean	SD	Range
PPH	Gestational Age	39.0	1.0	35.60–41.10
	Woman’s Weight	88.3	20.4	56.60–148.30
	Newborn’s Weight	3.3	0.5	2.40–4.40
	APGAR Score	8.9	0.4	7.00–9.00
Non-PPH	Gestational Age	38.8	1.1	35.50–41.60
	Woman’s Weight	79.9	14.2	53.50–119.20
	Newborn’s Weight	3.2	0.4	2.20–4.10
	APGAR Score	8.9	0.2	8.00–9.00

PPH: Postpartum Hemorrhage, Non PPH: without postpartum Hemorrhage.

**Table 3:** Rate of PPH per Year from 2009–2015.

Year	Total Number of Deliveries	Number and (Percent) of Women with PPH
2009	3752	133 (3.54)
2010	3639	194 (5.33)
2011	3697	196 (5.30)
2012	3817	205 (5.37)
2013	3967	207 (5.22)
2014	4271	230 (5.39)
2015	3943	280 (7.10)

**Table 4:** Duration of Third Stage of Labor in Subgroups for Women with PPH and Women without PPH.

Groups	Subgroups	*n*	Mean	SD	Minimum	Maximum
PPH	SSC only	11	11.9	11.4	2.00	29.00
	SSC + BF	28	11.6	12.4	1.00	65.00
	No SSC/no BF	27	18.3	19.3	1.00	1.00
	Total	70	14.6	15.7		
Non-PPH	SSC only	7	5.9	3.2	3.00	11.00
	SSC + BF	31	6.1	4.2	1.00	18.00
	Total	38	6.0	4.0		

**Table 5:** Amount of EBL in Women with PPH and Women without PPH.

Groups	Subgroups	*n*	Mean	SD	Minimum	Maximum
PPH	SSC only	11	836.4	518.7	500	2000
	SSC + BF	29	728.3	445.7	500	2000
	No SSC/No BF	33	1149.2	676.9	500	3000
	Total	76	934.8	592	1500	7000
Non-PPH	SSC only	7	131.4	37.6	100	200
	SSC + BF	30	210.0	78.1	100	350
	No SSC/No BF	33	228.8	81.0	100	400
	Total	73	209.9	79.4	300	950
